# The microbiome and autoimmunity: a paradigm from the gut–liver axis

**DOI:** 10.1038/cmi.2018.7

**Published:** 2018-04-30

**Authors:** Bo Li, Carlo Selmi, Ruqi Tang, M E Gershwin, Xiong Ma

**Affiliations:** 1 Division of Gastroenterology and Hepatology, Key Laboratory of Gastroenterology and Hepatology, Ministry of Health, State Key Laboratory for Oncogenes and Related Genes, Renji Hospital, School of Medicine, Shanghai Jiao Tong University, Shanghai Institute of Digestive Disease, 200001 Shanghai, China; 2 Division of Rheumatology and Clinical Immunology, Humanitas Research Hospital, Rozzano, Italy; 3 BIOMETRA Department, University of Milan, Milan, Italy; 4 Division of Rheumatology, Department of Medicine, Allergy and Clinical Immunology, University of California at Davis, Davis, CA USA

## Abstract

Microbial cells significantly outnumber human cells in the body, and the microbial flora at mucosal sites are shaped by environmental factors and, less intuitively, act on host immune responses, as demonstrated by experimental data in germ-free and gnotobiotic studies. Our understanding of this link stems from the established connection between infectious bacteria and immune tolerance breakdown, as observed in rheumatic fever triggered by *Streptococci* via molecular mimicry, epitope spread and bystander effects. The availability of high-throughput techniques has significantly advanced our capacity to sequence the microbiome and demonstrated variable degrees of dysbiosis in numerous autoimmune diseases, including rheumatoid arthritis, type 1 diabetes, multiple sclerosis and autoimmune liver disease. It remains unknown whether the observed differences are related to the disease pathogenesis or follow the therapeutic and inflammatory changes and are thus mere epiphenomena. In fact, there are only limited data on the molecular mechanisms linking the microbiota to autoimmunity, and microbial therapeutics is being investigated to prevent or halt autoimmune diseases. As a putative mechanism, it is of particular interest that the apoptosis of intestinal epithelial cells in response to microbial stimuli enables the presentation of self-antigens, giving rise to the differentiation of autoreactive Th17 cells and other T helper cells. This comprehensive review will illustrate the data demonstrating the crosstalk between intestinal microbiome and host innate and adaptive immunity, with an emphasis on how dysbiosis may influence systemic autoimmunity. In particular, a gut–liver axis involving the intestinal microbiome and hepatic autoimmunity is elucidated as a paradigm, considering its anatomic and physiological connections.

## Introduction

The incidence of autoimmune and inflammatory diseases has been increasing worldwide, along with an earlier diagnosis and increased physician awareness. More importantly, changes in environmental factors, such as a modern lifestyle, dietary habits, antibiotic use and hygiene, are hypothesized to have a critical role,^1^ as ideally represented by the largely incomplete concordance in autoimmune and chronic inflammatory diseases observed in monozygotic twins,^2^ in whom differences in the gut microbiome have also been emphasized.^3,4^ Indeed, human intestinal mucosal sites represent the sites that are most influenced by the surrounding environment, and millions of microbial residents have emerged as a unique organ that constantly shapes host immunity and metabolism. Recently, perturbed microbial composition and function, termed ‘dysbiosis’, has been associated with autoimmune diseases, particularly, rheumatoid arthritis (RA), type 1 diabetes (T1D), multiple sclerosis (MS) and autoimmune liver disease (AILD). Manipulation of germ-free and gnotobiotic animal models also sheds some light on how specific changes in the microbiome affect host autoimmunity.^5^

The mechanistic links between the microbiome and autoimmune diseases remain largely unknown. It seems plausible to draw an analogy from how infectious bacteria disrupt immune tolerance (for example, rheumatic fever triggered by *Streptococci*), such as molecular mimicry, epitope spread and bystander effects.^6,7,8^ Indeed, it has been demonstrated that apoptosis of intestinal epithelial cells in response to microbial infection enables the presentation of self-antigens, resulting in differentiation of autoreactive Th17 cells and other T helper cells.^9^ By using a spontaneous model of autoimmune uveitis, Horai *et al.* ^10^ have elegantly demonstrated that retina-specific T cells are first activated in the intestine by commensal microbes and subsequently evoke autoimmune attack in the immune-privileged eye site. In addition, a recent study involving three infant cohorts showed that children from districts with a higher prevalence of autoimmune diseases are dominated by bacterial species that produce less immunogenic lipopolysaccharide (LPS)^11^ and that different microbiome-derived LPSs display different structures and immunogenic functions, which may influence early-life immunological education and account for the variable autoimmune disease predisposition among individuals.^11^ One may speculate that the associations between commensal microbiome and host autoimmunity are rather complex and multifactorial. Apparently, harnessing the intestinal microbiome to control and prevent autoimmune diseases or using this information to diagnose and stratify patients still requires a more comprehensive characterization of the disease-related microbiome and a deeper understanding of its causality.

Thus, this review will first provide a basic overview of the interactions between the intestinal microbiome and the immune system, with an emphasis on how these interactions affect host autoimmunity locally and systemically, which might underlie the human microbiome studies described in this article. In particular, a gut–liver axis involving intestinal microbiome and autoimmune liver diseases will be elucidated separately, given the special anatomic and physiological relationships of liver, intestine and its microbial contents. We will not discuss the bacterial species observed in other sites, such as the skin or genitalia.

### The gut microbiome and immunity

Mounting evidence points towards a potential involvement of immune dysregulation at mucosal sites during the initiation and progression of autoimmune diseases. One hypothesis is that in the settings of intestinal inflammation, impairment of the gut barrier results in bacterial translocation, which then stimulates immune reactions in distant organs. Alternatively, immune cells are abnormally skewed by dysbiosis, as observed for Th17 polarization. Indeed, Th17 cells are most abundant in the *lamina propria* of the intestine, where they secrete the pro-inflammatory cytokines interleukin (IL)-17A, IL-17F and IL-22 to enforce gut barrier integrity and defense against pathogens.^12^ It is now widely accepted that understanding the complex interactions between the microbiome and immune system will be crucial to defining and treating or reversing the pathogenesis of chronic autoimmune diseases.

*Interaction between the microbiome and innate immunity*

Innate immune cells are strategically distributed at the host–microbiome interface at mucosal sites and constitute the first line to sense the components or products of microorganisms and to transduce signals into the host, eliciting responses that may in turn alter the composition and function of the microbiome.^5^ Dysbiosis has been observed in several mouse models of innate immune deficiency, including mice that lack the genes MyD88,^13^ Toll-like receptor 5 (TLR5)^14^ and nucleotide-binding oligomerization domain-containing protein 2 (NOD2).^15,16^ TLRs and NOD-like receptors (NLRs) are both pattern recognition receptors (PRRs) by which the host senses conserved components of the microbiome. The absence of these innate immune receptors undermines defense against pathogens, predisposing the tissue to spontaneous inflammation. For instance, mice that are deficient in TLR5 develop features of metabolic syndrome correlated with an altered microbiome.^14^ The absence of NOD2, an intracellular PRR for bacterial peptidoglycans, elicits transmissible colitis and colitis-associated carcinogenesis in mice, possibly due to an impaired restriction of *Bacteroides vulgatus*.^15,17^

Recent studies have revealed a central role of the inflammasome pathway and interleukin-18 (IL-18) in orchestrating host–microbial crosstalk. Inflammasomes are multi-protein complexes consisting of NLR proteins, ASC and caspase-1. Once assembled, inflammasomes become activated via auto-cleavage of pro-caspase-1, which then processes IL-1β and IL-18. Levy *et al.* ^18^ revealed that the microbiome-associated metabolites taurine, histamine and spermine can induce NLRP6 inflammasome activation, IL-18 production and subsequent anti-microbial peptide (AMP) generation, which is key for the integrity of the gut barrier and commensal colonization. This inflammasome modulation by the microbiome has recently been confirmed by the discovery that intestinal colonization with the protozoan *Tritrichomonas musculis* also activates the epithelial inflammasome to release IL-18, which promotes Th1 and Th17 immunity driven by dendritic cells.^19^

In addition to phagocytosis, the formation of neutrophil extracellular traps (NETs), a web-like DNA structure with numerous anti-microbial proteins and proteolytic enzymes, is another process that enables neutrophil to capture and eradicate bacteria. Emerging evidence has indicated that NETs are associated with noninfectious pathologic conditions, including the autoimmune diseases RA and systemic lupus erythematosus (SLE).^20,21,22^ Moreover, the interaction between NETs and dysbiosis in the setting of inflammatory and autoimmune diseases has been of particular interest. Vong *et al.* ^23^ reported the differential capacity of intestinal microbes to elicit NETs. Accordingly, the probiotic *Lactobacillus* spp. dampen the ability of neutrophils to form NETs, whereas enteropathogenic *E. coli* exhibit an increased capacity to mobilize the neutrophil oxidative burst and activate NETs, suggesting an innate connection between dysbiosis and neutrophil function.^24,25^ In addition, NETs have been associated with sites beyond the intestine, such as RA-related autoimmunity in the lungs.^21^ The frequency of NET complexes is correlated with the sputum microbial diversity and an impaired capacity for neutrophil phagocytosis of bacteria in patients with chronic obstructive pulmonary disease (COPD).^25^ Whether the dominance of NETs precedes the disturbance of mucosal microbiota or the pathogenic microbes elicit excessive NET production and how they interact to initiate and maintain the chronic inflammation require further investigation.

Except for the general recognition of bacteria by PRRs, the microbiome can signal through metabolites and affects host transcription in the intestine or in distal organs.^26^ Indeed, short-chain fatty acids (SCFAs), the major fermentation products that are generated from commensal microbiome degrading dietary fiber, are capable of regulating cell functions either by modification of histone deacetylase (HDAC)^27^ or by activation of ‘metabolite-sensing’ G-protein-coupled receptors (GPCRs).^28^ For example, SCFAs derived from a high-fiber diet protect mice against colitis by binding to GPR43 and GPR109 on colonic epithelium cells, which leads to NLRP3 inflammasome activation and IL-18 release.^29^ Deprivation of dietary fiber renders the gut microbiome mucus-degrading, which further enhances mucosal susceptibility to the pathogen *Citrobacter rodentium*.^30^ In addition to SCFAs, catabolism of the amino acid tryptophan to indole derivatives by commensal bacteria is also well positioned to balance gut homeostasis. Zelante *et al.* ^31^ have shown that a subset of *Lactobacilli* utilizes tryptophan to produce indole-3-aldehyde (IAID), which then acts as a ligand of aryl hydrocarbon receptor (AHR). Activation of AHR on innate lymphoid cells (ILCs) promotes its production of IL-22 and thus provides colonization resistance to *Candida albicans*. Manipulation of the feedback control of AHR signaling cytochrome P4501 (CYP1) enzymes results in a loss of AHR-dependent ILC3 and Th17 cells, which can be reversed by supplementation of AHR ligands in the diet.^32^ Recently, impaired tryptophan metabolism by the microbiome due to a deficiency in the *CARD-9* gene has also been associated with susceptibility to colonic inflammation.^33^ Other metabolites, such as inosine, may be remodeled by *Lactobacillus reuteri*, which then suppress the autoimmunity caused by Treg deficiency.^34^

Thus, the interactions between microorganisms and the host extend well beyond classic immune cells. ILCs are a newly discovered arm of the innate immune system that are enriched at mucosal sites, and the ILC3 subset is characterized by RORγt expression and is most related to the commensal microbiome. Intestinal lymphoid tissue colonization by commensal bacteria is beneficial for host–microbial mutualism, mainly by inducing DC-derived IL-10 and group 3 ILC-derived IL-22.^35^ However, depletion of ILCs results in a defective containment of these commensal bacteria and promotes systemic inflammation.^36^ IL-22 secreted by ILC3 has received particular attention in gut homeostasis because of its pleiotropic roles in AMP release, mucus production, intestinal epithelial regeneration and colonization resistance to pathogens.^31,37^ Moreover, lymphotoxin (LT)-α and LT-β expressed by ILC3s contribute to T cell-dependent and T cell-independent IgA induction.^38^ In contrast, the dynamics of the intestinal microbiome have been suggested to impact the epigenetic regulation and gene expression of ILC.^39^ Flagellin sensed by CD103+ myeloid cells promotes the production of IL-22 by ILCs through IL-23.^40^ Upon microbial recognition, IL-1β produced by intestinal macrophage drives granulocyte-macrophage colony-stimulating factor secretion by ILC3, which then acts on DCs to adjust intestinal T cell tolerance to commensals.^41^ However, instead of conventional CD103+ dendritic cells (cDCs), Longman *et al.* ^42^ showed that monocyte-derived CX3CR1+ cells are more efficient in providing IL-23 and IL-1β, thus supporting IL-22 production by ILC3. Intriguingly, it has recently been proposed that ILC3s expressing class II MHC can induce apoptosis of commensal microbiome-reactive CD4+ T cells.^43^ Similar to medullary thymic epithelial cells, ILC3 presents commensal antigens to CD4+ T cells in the intestine to maintain host tolerance to symbiotic bacteria.

Other non-classical lymphocytes, including γδ T cells, natural killer T (NKT) cells, and mucosal-associated invariant T (MAIT) cells, are also of particular interest in the host–microbiome crosstalk, given their abundance in the intestine and liver. A previous study has shown that γδ T cells can be directly activated by specific microbial colonization and function as an innate source of IL-17.^44^ Recently, a unique liver-resident γδ T cell subset that predominantly produces IL-17 A has been identified, and homeostasis of these liver γδ T cells is maintained by the gut microbiome in a lipid antigen/CD1d-dependent manner.^45^ Regarding CD1d-dependent invariant NKT (iNKT) cells, early-life contact with commensal microbes is key for tolerance establishment. The iNKT cells in germ-free mice tend to accumulate in colonic lamina propria and exacerbate experimental IBD and asthma,^46^ whereas recognition of sphingolipids derived from *Bacteroides fragilis* reduces the proliferation of these destructive NKT cells and thus ameliorates the inflammatory disease.^47^ Of note, mouse infection by *N. aromaticivorans* induces anti-mitochondrial antibody (AMA) and T cell-mediated small bile duct damage characteristic of primary biliary cholangitis (PBC), and initiation of this autoimmune attack is dependent on the microbial activation of NKT cells.^48^ Likewise, MAIT cells are restricted by a highly conserved class Ib MHC molecule, the MHC class I-related protein 1 (MR1).^49^ MAIT cells are also important in the host–microbiome crosstalk, with the bacterial riboflavin metabolite 5-A-RU being its potent ligand. The absence of MAIT cells in germ-free mice underlies the essence of the commensal microbiome in MAIT cell development.^49^ Intrahepatic MAIT cells have been found to actively respond to bacterial-exposed biliary epithelial cells, indicating their potential role in the mucosal homeostasis of bile ducts.^50^

*Interaction between the microbiome and adaptive immunity*

IgA is the most abundant secretory Ig isotype in the gut and can be produced in both T cell-independent and in a T cell-dependent manner.^51^ Commensal microbial stimulation is required for intestinal IgA responses, with good examples being *segmented filamentous bacterium* (SFB) and *Alcaligenes*.^52,53^ However, the intestinal microbiome in low-IgA mice has been shown to degrade the secretory component of secretory IgA and IgA itself, which may account for its susceptibility to colitis.^54^ In a reciprocal fashion, IgA has a fundamental role in mucosal defense by coating and entrapping microorganisms and, moreover, immobilizing the microbiome via downregulating the expression of flagella-related genes.^55^ A fascinating working hypothesis states that IBD might be preferentially initiated by commensals with high IgA coating, as suggested by the novel techniques in sorting and sequencing immunoreactive pathosymbionts (termed IgA-seq).^56^ An IgA-coated *Escherichia coli* was found to be specifically enriched in patients with Crohn’s disease and concomitant spondyloarthritis, colonization of which was capable of inducing Th17 mucosal immunity.^57^ Coating by IgA enables the translocation of non-invasive microbes, facilitating antigen presentation and subsequent production of antigen-specific IgA.^58^ These IgA, which have undergone somatic hypermutation and affinity maturation, further bind to and select for particular microbes. Therefore, IgA functions to shape and maintain the microbial community. Other immune cells, such as Treg cells and follicular helper T (Tfh) cells, also impinge on gut microbial diversity through IgA selection in Peyer patches.^59,60^

Germ-free (GF) and antibiotic-treated mice are partially defective in adaptive immunity, characterized by a paucity of intestinal Th17 and Treg cells and a skewing toward Th2. Ivanov *et al.* ^61^ have shown that monocolonization by SFB in mice is sufficient to induce intestinal Th17 cells. Notably, SFB-dependent Th17 cells exhibit a dichotomous effect because they confer resistance to enteropathogens in mice, but potentiate autoimmune inflammation in murine models of multiple sclerosis (MS) and rheumatoid arthritis (RA).^62,63^ Moreover, neonatal colonization of SFB in mice and its associated IL-17 signaling has been shown to impact antinuclear antibody production and resultant systemic autoimmunity in adult life.^64^ Subsequent investigations into SFB have suggested that Th17 cell responses to SFB are at least partially antigen-specific.^65^ It has been reported that MHC-II-dependent presentation of SFB antigens by conventional DCs drives mucosal Th17 cell differentiation.^66^ In addition, direct adhesion of SFB to the ileal epithelium induces serum amyloid A proteins 1 and 2 (SAA1/2). In parallel, SFB activates ILC3 to produce IL-22, which further promotes epithelial SAA production.^67^ However, how SAA acts on Th17 cells is unclear. It is hypothesized that CX3CR1+ myeloid cells may respond to SAA and secrete cytokines to promote the polarization of Th17 cells as well as IL-22 production by ILC3.^42,68^ Nonetheless, SFB has not yet been identified in human intestines, whereas *Bifidobacterium adolescentis*, a human-derived symbiotic bacterial species, acts as an identically potent inducer of Th17 cells via a non-SFB mechanism in murine intestines.^69^ In contrast, depletion of *Lactobacillus murinus* by high salt consumption may also lead to Th17 dysregulation and autoimmune disorders, which can be rescued by supplementation with *L. murinus*.^70^ The same reduction of *Lactobacillus* spp. and an increase in Th17 cells due to high salt intake have also been observed in a pilot human study, giving rise to the connection between the diet and the gut–immune axis.

Immunity at mucosal surfaces requires a delicate balance to resist pathogenic infections and maintain tolerance to commensals. Intestinal homeostasis is maintained by Treg cells, which prevent aberrant immune responses toward dietary antigens and the commensal microbiome, thus halting the initiation of immunopathology. Intestinal Treg cells are induced and maintained by certain members of the commensal microbiome,^71^ as supported by the observation that the *Bacteroides fragilis*-derived polysaccharide, PSA, can restore immunologic deficiency in GF mice.^72^ PSA is a symbiotic factor with a capacity to mediate the conversion of CD4+ T cells into IL-10-producing Tregs and to ameliorate mucosal inflammation in mice.^73,74^ This immunomodulatory effect requires two IBD-associated genes, ATG16L1 and NOD2, to activate a non-canonical autophagy pathway, which might account for the defective Treg responses in individuals with such risk genes.^75^ Moreover, the anti-inflammatory effect of PSA has been extended to extraintestinal autoimmune mouse models, such as multiple sclerosis.^76^ The work of Atarashi *et al.* ^77^ demonstrates the capability of a selected consortium of *Clostridia* strains in inducing Treg cells in the murine intestine, in support of the idea that the tolerogenic cell type is largely conferred by local microbial communities.^78^ Mechanistically, this microbial induction of Tregs may be mediated by SCFAs, particularly butyrate, through HDAC inhibition and consequent histone H3 acetylation of the *Foxp3* gene.^27^ Alternatively, SCFAs stimulate the proliferation of Treg cells by activating G-protein-coupled receptors such as GPR43.^28^

Recent studies further add to the complexity of the symbiont-induced Tregs and Th17 cells. Two groups identified a subset of Tregs that generally lack NRP1 and Helios and, surprisingly, express RORγt, a transcription factor that is thought to antagonize FoxP3 and promote Th17 cell differentiation.^79,80^ This FoxP3+ RORγt+ Treg subtype expresses high levels of IL-10 and CTLA-4 and exhibits enhanced suppressive capacity in experimental colitis.^81^ In parallel, another population of intestinal Treg cells driven by GATA3 is induced by epithelium-derived IL-33.^82^ How the microbiome and other tissue-derived factors regulate the balance between GATA3-expressing and RORγt-expressing Treg cells remains unknown, but the latter cells are not locally confined. Shifting the Treg balance in the intestine by various environmental factors may have systemic implications leading to the perpetuation of autoimmune injury and chronic inflammation.

Interestingly, it has been suggested that the microbiota might also have an essential role in lymphopenia-associated autoimmunity, which could account for the paradoxical concurrence of autoimmunity and immunodeficiency within an individual. During lymphopenia, peripheral T cells undergo a process termed ‘homeostatic proliferation’ to maintain the immune system while giving rise to the possibility of an aberrant expansion of autoreactive clones.^83,84^ Under this circumstance, a two-hit model driven by commensal microbiota has been proposed to explain the pathogenesis of lymphopenia-associated colitis.^85^ First, the microbiota stimulates innate cell production of IL-6 via MyD88, which provides signals for the spontaneous proliferation of T cells. With the presence of microbiota, these T cells then proliferate in an antigen-specific manner and cause colon inflammation. However, an inconsistently normal or even increased T cell homeostatic proliferation has been observed in MyD88- and several TLR-deficient mouse models.^86^ Recently, Eri T *et al.* ^87^ have shown that conventional T cells transferred into athymic mice will proliferate and differentiate into a distinctive PD-1+CXCR5−/dim T cell subset, acting as Tfh cells to promote B cell autoantibody production. Depletion of commensal microbiota by antibiotics inhibits differentiation and ameliorates systemic autoimmunity.^87^ Collectively, an indispensable role of the microbiota in lymphopenia-induced autoimmunity has been demonstrated in several models, although the underlying detailed mechanisms are not entirely understood.

### The gut microbiome and autoimmune diseases

**Figure 1:**
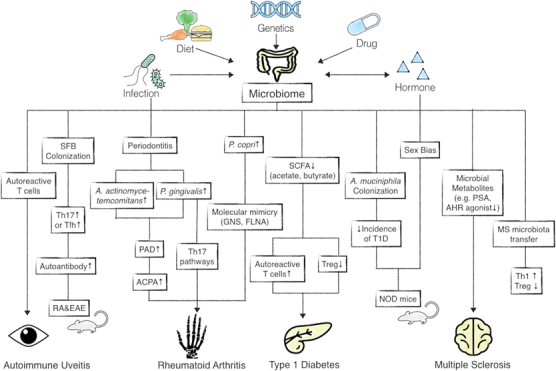
The proposed link between the gut microbiome and systemic autoimmune diseases such as rheumatoid arthritis (RA), type 1 diabetes (T1D) and multiple sclerosis (MS). PAD, peptidylarginine deiminase; ACPA, anti-citrullinated protein antibodies; GNS, *N*-acetylglucosamine-6-sulfatase; FLNA, filamin A; SCFAs, short-chain fatty acids; NOD, non-obese diabetes; PSA, polysaccharide derived from *Bacteroides fragilis*; AHR, aryl hydrocarbon receptor; SFB, *segmented filamentous bacterium*; Tfh, follicular helper T cell; EAE, experimental autoimmune encephalomyelitis.

Multiple lines of evidence have linked dysbiosis to barrier autoimmunity and beyond, particularly in the setting of IBD, rheumatoid arthritis, type I diabetes and multiple sclerosis (Figure 1).^88^ First, the altered composition and function of the microbiome was assessed in different animal models of autoimmune diseases, mainly using germ-free and gnotobiotic mice colonized with a defined microbe. In parallel, human observational studies have been conducted in patients with autoimmune diseases, using 16s ribosomal RNA (rRNA) sequencing, metagenomic sequencing and metabolomics analysis. As a result, human studies have led to large amounts of descriptive data for which mechanistic interpretations should be obtained from experimental models; however, therapeutic attempts are already ongoing in clinical settings.

*Rheumatoid arthritis (RA)*

Environmental factors are likely to have a pivotal role in the development of RA in genetically prone individuals,^89^ and the microbiome is a presumed culprit because germ-free mice are protected against experimental arthritis.^63,90^ The introduction of SFB in GF K/BxN mice restores the production of autoantibodies and arthritis by inducing Th17 cells in the intestinal lamina propria.^63^ One possible mechanism for this phenomenon is that the commensal microbiome induces DC and macrophage production of pro-inflammatory cytokines such as IL-1β and IL-6, which facilitate Th17 differentiation and the onset of arthritis.^91^ IL-10-producing Breg cells are induced in mice by the same signals, IL-1β and IL-6, triggered by the microbiome,^92^ thus supporting a dual role of the microbiome in establishing both pro-inflammatory and regulatory immune responses. Intriguingly, Wu and colleagues^93^ recently proposed a previously unknown conception that gut commensals, exemplified by SFB, might selectively expand Th17 cells expressing dual TCRs, which recognize both SFB antigens and self-antigens. These Th17 cells are recruited though the CCL20–CCR6 axis to the lung, where they trigger RA-related lung pathology. This dual TCR mechanism, independent of molecular mimicry or bystander activation, has yielded new sights into how the commensal microbiota modulate host autoimmunity.

Patients with new-onset RA manifest a microbiota enriched for the pathobiont *Prevotella copri*, whereas patients with established RA have significantly lower abundance, possibly due to the impact of treatment^94^ and the presence of IgG or IgA antibodies that are reactive to *P. copri* in RA subgroups.^95^ Two autoantigens, *N*-acetylglucosamine-6-sulfatase (GNS) and filamin A (FLNA), have shown marked sequence homology to epitopes of gut microbes, such as *Prevotella* sp., further supporting the molecular mimicry theory in RA.^96^ Recently, a metagenome-wide association study comparing RA patients and healthy controls has confirmed a perturbed microbiome in the intestine, dental region and saliva, which was partially resolved after RA treatment.^97^ A discrete concordance exists between the gut and oral microbiomes in individuals with RA, suggesting an overlap in their pathogenicity at different body sites, particularly considering the proposed link between periodontal infection by *Porphyromonas gingivalis* and RA. Periodontitis is more prevalent in RA and is associated, in particular, with high levels of anti-citrullinated protein antibodies (ACPA).^98^ Mechanistic studies in mouse models have revealed an involvement of Th17-related pathways in this autoimmune arthritis model driven by periodontal pathogens.^99^ There is increasing evidence that the link between these two diseases is due to the unique peptidylarginine deiminase (PAD) enzyme expressed by *P. gingivalis*, which specifically citrullinates peptides from key autoantigens of RA and thus breaks immune tolerance.^100^ An alternative pathogenic candidate may be *Aggregatibacter actinomycetemcomitans*, which triggers hypercitrullination in host neutrophils.^101^

*Type I diabetes (T1D)*

A significant contribution of non-genetic factors has been recognized in the development of T1D since a marked discrepancy exists between individuals carrying T1D-associated HLA risk alleles and those who develop the disease.^102^ A multi-hit model for T1D has recently been suggested by a prospective study of 33 infants who were genetically predisposed to T1D, showing that changes in the gut microbiome occur after seroconversion to autoantibody positivity but before the onset of T1D.^103^ The notion that the gut microbiome may be involved in T1D pathogenesis through the connection between the gut and the pancreas has also been well established in murine models.^13,104,105,106^ For example, non-obese diabetic (NOD) mice lacking MyD88, a downstream adaptor for multiple TLR-related signaling pathways, are protected against diabetes, but this effect was abrogated in the absence of commensal bacteria.^13^ Transfer of the gut microbiome from these diabetes-protected MyD88-deficient NOD mice shaped mucosal immunity and further delayed the onset of diabetes in the recipients.^107^ The possible involvement of molecular mimicry by the microbiome in immune tolerance breakdown in T1D^108^ is supported by the development of accelerated T1D in MyD88-/- mice due to an altered intestinal microbiome encompassing a microbial peptide mimic of IGRP, an islet-specific autoantigen.^108^ Conversely, expression of certain MHC-II alleles can protect NOD mice from autoimmune insulitis, which seems to be mediated by the intestinal microbiota.^109^ More importantly, this protection occurs during a critical early window of ontogeny, highlighting the possibility of modulating the microbiota at an early stage to prevent autoimmune diseases. In addition, gut microbial metabolites also appear to be protective, and feeding NOD mice a combined acetate- and butyrate-yielding diet prevents the development of T1D, mainly through autoreactive T cells limited by acetate and Treg cells boosted by butyrate.^110^ Very recently, it was demonstrated that the transfer of a single symbiont, *Akkermansia muciniphila*, to NOD mice, instead of the whole microbial community, could significantly lower the incidence of T1D, with its multiple immunologic and metabolic signaling remodeling effects.^111^ In agreement with these experimental data, a series of cohort studies have shown a specifically altered microbiome profile in individuals with preclinical T1D, including a sharp decrease in microbiome diversity, low community stability, a dominating abundance of the *Bacteroides* genus, fewer *Bifidobacterium* species, and a dearth of butyrate-producing and lactate-producing species.^103,112,113^

Although sexual dimorphism is another prominent feature of many autoimmune diseases that predominantly affect women, T1D is one of the few exceptions. NOD mice are characterized by spontaneous, immune-mediated loss of pancreatic β cells mimicking T1D, while displaying a strong gender bias. Strikingly, germ-free conditions reverse the differences of T1D incidence commonly observed between male and female NOD mice and transfer of the male microbiome to female mice is protective against T1D, possibly via protective testosterone levels.^114^ However, another group has reported that hormones contribute reciprocally to the sex-based microbial differences.^114^ The higher testosterone level in adult NOD male mice selectively enriches organisms such as SFB and an *E. coli*-like species, which in turn upregulate host testosterone. Collectively, these two factors, that is, the microbiome and hormones, might work as a feedback loop to influence the incidence of autoimmune diseases other than T1D.^115^

*Multiple sclerosis (MS)*

The initiation of autoimmunity in individuals who are genetically predisposed to MS has been attributed to environmental factors, particularly microbial infection, based on data from murine models of experimental autoimmune encephalomyelitis (EAE).^116^ Earlier evidence from a spontaneous, relapsing-remitting mouse model of EAE has shown that the commensal microbiome is required to induce self-reactive B cells upon simulation with the autoantigen, myelin oligodendrocyte glycoprotein (MOG).^117^ Germ-free mice exhibit compromised EAE; however, colonization of *SFB* alone restores the disease severity comparable to conventionally raised mice.^62^ Conversely, administration of PSA derived from *B. fragilis* protects mice against central nervous system (CNS) demyelination and inflammation by tissue-specific expansion of CD4+Foxp3+Tregs expressing CD39.^76,118^ Intriguingly, it has recently been shown that the metabolism of dietary tryptophan into AHR agonists by the gut microbiome also seems to be involved in the gut–brain axis.^31,119^ These intestinal metabolites enter the systemic circulation and act on astrocytes to alleviate CNS inflammation.^119^ Consistently, decreased levels of circulating AHR agonists are observed in individuals with MS, and cross-sectional human studies of relatively small cohorts suggest a distinct alteration in the MS gut microbiome compared with healthy controls.^120,121^ A study of 60 relapsing–emitting MS cases has reported an increased abundance of *Methanobrevibacter* (Archaea) and *Akkermansia* and a reduction in *Butyricimonas*.^120^ Notably, two recent studies, by combining cohort analysis and *in vitro* and *in vivo* experiments, further propelled the mechanistic study.^122,123^ Although no overt difference in the overall microbial profile was found in 34 monozygotic twin pairs who were discordant for MS, when transplanted into the spontaneous mouse model of EAE, the gut microbiota derived from twin bearing MS aggravated the disease compared with the healthy twin-derived microbiota.^122^ In a study by Cekanaviciute *et al.*,^123^ the microbiomes of 71 untreated MS patients were analyzed. By subsequently exposing PBMCs to bacterial extracts and monocolonizing mice with specific species, they showed that MS-associated bacteria indeed shifted T cells toward a pro-inflammatory profile. For instance, *Akkermansia muciniphila*, which was increased in MS patients, boosted Th1 cell differentiation. In tandem with the study by Berer *et al.*,^122^ the compromised IL-10 production by Treg cells in mice colonized with MS patient-derived microbiota was also addressed.^123^ These two independent studies, by defining the potential mechanisms of T cell autoimmunity induced by commensal microbiota, have improved our understanding of the causal link between the microbiome and autoimmune disease.

### Gut microbiome and liver autoimmunity

**Figure 2:**
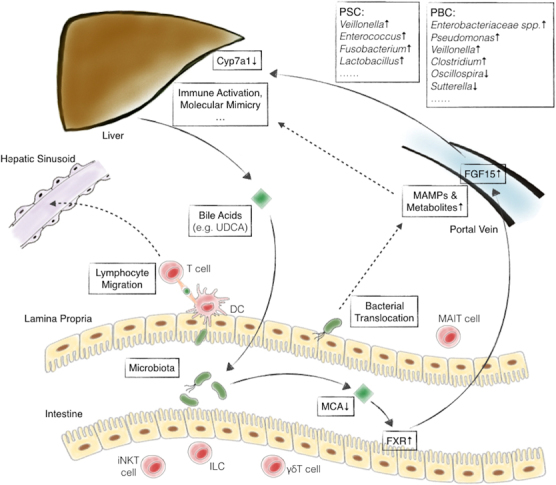
The paradigm of gut microbiome involvement in liver autoimmunity. On the basis of the gut–liver axis, bacterial translocation, migration of gut-primed lymphocytes to the liver, bile acids and nuclear receptor signaling are involved in PBC and PSC pathogenesis. Cyp7a1, cholesterol 7 alpha-hydroxylase; Fgf15, fibroblast growth factor 15; MAMPs, microbe-associated molecular patterns; UDCA, ursodeoxycholic acid; MCA, muricholic acid; FXR, farnesoid X receptor; PSC, primary sclerosing cholangitis; PBC, primary biliary cholangitits; iNKT cell, invariant natural killer T cell; ILC, innate lymphoid cell; γδT cell; MAIT cell, mucosal-associated invariant T cell.

With 70% of its blood supply derived from the portal vein, the liver is physiologically exposed to gut-derived microbial components and metabolites, and intestinal dysbiosis has been associated not only with liver diseases, including non-alcoholic fatty liver disease (NAFLD), but also inflammatory, fibrotic or cholestatic conditions.^124^ Not surprisingly, this gut–liver axis has also been implicated in autoimmune liver disease (Figure 2), as well represented by primary sclerosing cholangitis (PSC) and primary biliary cholangitis (PBC).^125,126^

*Bacterial translocation and immune activation in the liver*

As mentioned above, the intestinal mucosal immune system, particularly mesenteric lymph nodes (MLNs), compartmentalizes the commensal microbiome. In addition to MLNs, the liver acts as a second firewall to eradicate bacteria that have overcome intestinal barriers.^127^ Notably, this function seems to be compromised in chronic liver diseases, but how Kupffer cells fail to clear intestinal microbes during the course of liver disease remains unclear. In this regard, the liver is not only a recipient but a filter of gut-derived agents with Kupffer cells, hepatic sinusoidal endothelial cells (HSECs) and biliary epithelial cells (BECs) all expressing PRRs, and they are able to sense microbe-associated molecular patterns (MAMPs) such as bacterial LPS, peptidoglycans, flagellin and bacterial DNA, among other ligands. The excessive immune responses elicited by these MAMPs are thought to lead to liver injury and fibrosis, and consistent with this hypothesis, a recent study has described a gut–vascular barrier (GVB) that controls microbiome translocation, which is impaired in celiac disease patients with unexplained elevated serum transaminases.^128^ As a result of these changes, bacterial translocation might drive extraintestinal inflammation, with hepatocytes and cholangiocytes being the vulnerable cell types.

The ‘leaky gut’ hypothesis has been exemplified in NAFLD as well as in PBC and PSC. For instance, a deficient NLRP6 and NLRP3 inflammasome aggravates liver steatosis as a consequence of TLR4 and TLR9 agonists in the portal circulation.^129^ Knockout of CX3CR1 in mice increases endotoxin in portal serum and promotes steatohepatitis.^130^ Bacterial lipoteichoic acid is detected around the damaged bile ducts in PBC, and chronic bacterial exposure in normal mice leads to autoantigen production and subsequent cholangitis that mimics PBC.^131^ In addition, perinuclear antineutrophil cytoplasmic antibodies (p-ANCAs) detected in PSC may target beta-tubulin isotype 5 (TBB-5) as an autoantigen as well as an evolutionary bacterial protein, FtsZ,^132^ giving rise to the hypothesis of an immune cross-reactivity to gut microorganisms. In humans, it is likely that genetic polymorphisms that impair immune responses to the gut microbiome are partially responsible for liver diseases. Indeed, GWASs have established several genetic susceptibility loci shared by PSC and IBD, including CARD-9, fucosyltransferase 2 (FUT-2) and macrophage-stimulating protein 1 (MST-1), among others.^133,134,135,136^ Proteins encoded by these genes are intimately involved in innate and adaptive immunity, as illustrated by the fucosylation of intestinal epithelial cells by FUT-2, which also maintains host–microbiome symbiosis. Patients with PSC carrying FUT-2 variants have a different microbial pattern in the bile and colon and, more importantly, are associated with an increased frequency of biliary infections and incidence of dominant stenosis.^137^ CARD-9, an important downstream mediator of NOD2 and TLR signaling, has recently been related to IL-22 production and gut integrity. Mice deficient in CARD-9 display increased susceptibility to colitis due to a loss of bacterial species capable of metabolizing tryptophan into AHR agonists.^33^

*Migration of mucosal lymphocyte to the liver*

In parallel to the ‘leaky gut’ hypothesis, a ‘gut lymphocyte homing’ hypothesis has been proposed to explain this gut–liver axis associated with PSC and PBC. Mucosal lymphocytes are characterized by the expression of integrin α4β7 and chemokine receptor CCR9, which bind to the endothelial adhesion molecule MAdCAM-1 and chemokine CCL25, respectively. However, aberrant expression of MAdCAM-1 and CCL25 has been detected in hepatic sinusoidal endothelium in inflammatory liver disease, including PSC,^138^ leading to the hypothesis that the gut-primed T cells are abnormally recruited to the liver and may trigger autoimmune reactions upon recognition of the corresponding antigens.^139^ Indeed, in a mouse model of ovalbumin-induced colitis, T cells primed in the GALT by ovalbumin migrate to the liver and cause cholangitis when recognizing the same antigen, ovalbumin, on cholangiocytes.^140^ Considering the strong association between PSC and IBD and the defined role of the microbiome in IBD pathogenesis, it is tempting to assume that mucosal T cells are aberrantly activated by the commensal microbiome, further migrate to the liver and cross-react with antigens present in the liver. In line with this hypothesis, a recent sequencing study of the TCRβ chain has demonstrated that the gut-infiltrating and liver-infiltrating T cells in PSC-IBD patients are clonally related and may be able to recognize the same antigens.^141^ In addition to T cells, intrahepatic IgA-producing B cells are derived from gut-associated lymphoid tissues and are directed against commensal antigens.^142^ Given the increased serum levels of IgA often observed in chronic liver diseases, in particular PBC and PSC, this finding further highlights the potential involvement of the gut–liver axis in these immune disorders.

However, the mechanisms by which liver endothelial cells express gut-specific molecules remain elusive, although it has been speculated that the upregulation of hepatic vascular adhesion protein (VAP)-1 in PSC is responsible for the aberrant expression of liver MAdCAM-1.^125^ Cysteamine and other amines derived from gut bacteria and diets can enter the liver via the portal vein and act as substrates for VAP-1, a potent amine oxidase, leading to the generation of catabolites that induce MAdCAM-1 expression on HSECs.^143^

*The gut microbiome, bile acids and nuclear receptor signaling*

Apart from direct interaction with the immune system, relationships between the microbiome and bile acids (BA) are also representatives of the gut–liver crosstalk.^144^ Bile acids are primarily produced in hepatocytes and then metabolized into secondary bile acids by bacteria in the gut lumen. Early works have reported that the BA composition of GF mice is distinct from their conventionally raised (CONV-R) counterparts. Sayin *et al.* ^145^ have provided insights into the field by showing that CONV-R mice exhibit a decreased proportion of tauro-beta-muricholic acid (TβMCA) as well as a significantly reduced size of the BA pool. More importantly, MCAs are potent farnesoid X receptor (FXR) antagonists, and the gut microbiome can regulate BA synthesis in the liver by alleviating FXR inhibition in the ileum. Specifically, FXR activation in the intestine induces fibroblast growth factor 15 (FGF15) expression, which then reaches the liver and blocks 7-α-hydroxylase (CYP7A1), the rate-limiting enzyme in BA synthesis. Microbiome remodeling by antibiotics or antioxidants increases TβMCA and suppresses intestinal FXR signaling.^146,147^ Conversely, BA extensively modulates the microbiome directly or indirectly through activation of the innate immune system and further influences host physiology via the FXR and G-protein-coupled bile acid receptor TGR5.^148^ Indeed, the dynamics of host bile acids results in an altered composition of the gut microbiome in rats.^149^ Application of the FXR agonist obeticholic acid to cirrhotic rats partially prevents gut barrier dysfunctions and attenuates intestinal inflammation, leading to reduced bacterial translocation.^150^ Using GF and *Fxr-/-* mice, a recent study has demonstrated that diet-induced obesity and liver steatosis promoted by microbiome is dependent on its regulation of the BA profile and FXR signaling.^151^ Moreover, this altered FXR signaling can further shift the gut microbiome toward a more obesogenic configuration, and the circuitry of the gut microbiome, bile acids, and nuclear receptor signaling operates to impact host metabolism as well as hepatic pathogenesis.

*The gut microbiome and autoimmune liver disease*

Several sequencing studies have reported an altered gut microbial community in PSC and PBC, although there are discrepancies, in particular at the genus and species levels.

Our group has recently conducted 16s ribosomal RNA (rRNA) sequencing of the fecal microbiome of a PBC cohort, prior to UDCA use to avoid the confounding effect of bile acids. Moreover, a prospective study of 37 PBC patients before and after 6 months of UDCA treatment has also been performed.^152^ The gut microbiome in UDCA-naïve PBC exhibits a significantly reduced within-individual microbial diversity compared with healthy controls. This finding is inconsistent with a previous study using a relatively small PBC cohort, likely due to the influence of UDCA treatment.^153^ A microbial signature of PBC defined by 12 genera can be used to accurately distinguish PBC from controls in the validation cohort (with an AUC of 0.84–0.86). An increased unknown genus in the family of *Enterobacteriaceae* shows the strongest association with PBC, followed by *Pseudomonas*, *Veillonella* and *Clostridium*, whereas *Oscillospira* and *Sutterella* were decreased in the PBC cohort. Notably, among these 12 PBC-associated genera, the abundance of 6 was reversed after 6 months of UDCA treatment,^152^ in parallel with the treatment response, thus suggesting that the gut microbiome may be a potential target for the treatment of PBC.

Given the coexistance of IBD in up to 60–80% of PSC cases, the gut–liver axis has been linked to the pathogenesis of PSC. Published studies with sample sizes ranging from 11 to 85 patients have confirmed a distinct microbial profile in PSC.^154^ In general, the gut microbiome in PSC shows a marked deviation from healthy controls, characterized by a reduced microbial diversity (Table 1). Changes in the abundances of specific bacteria, such as *Enterococcus* and *Veillonella*, have been observed in different studies, and some might be used as biomarkers of PSC. Four of these studies analyzed mucosal biopsies,^155,156,157,158^ whereas others have focused on the fecal microbiome.^159,160,161,162^ The study by Kummen *et al.* ^161^ is the largest assessment of the fecal gut microbiome in PSC, resulting in the identification of a less diverse microbial profile in PSC, with *Veillonella* being a PSC-associated genus. However, the difference in the *Veillonella* genus in PSC was no longer significant when patients with liver cirrhosis were excluded, according to the study by Sabino *et al.* ^160^ In addition, Sabino *et al.* ^160^ identified three enriched genera in PSC, namely, *Enterococcus*, *Fusobacterium* and *Lactobacillus*, regardless of concomitant IBD or UDCA treatment. Among confounding factors, salazosulfapyridine (SASP) treatment should be excluded when analyzing the gut microbiome of patients with PSC, whereas data from mucosal microbiome are more heterogeneous than fecal ones, possibly due to the smaller sample size.^154^ Housing different PSC mouse models in germ-free facilities leads to contradictory conclusions because NOD.c3c4 mice develop cholangitis spontaneously with a specific gut microbiome, but they manifest a milder phenotype under GF conditions.^163^ In contrast, mdr2(−/−) mice under GF conditions exhibit exacerbated PSC-like manifestations with more severe cholestasis, liver fibrosis, a ductular reaction and ductopenia, without secondary bile acids.^164^ The administration of UDCA, a commensal bacterial metabolite, abrogates cholangiocyte senescence in vitro,^164^ and these discrepancies might be attributed to the distinct host genotype and pathogenic mechanisms in these two models.

**Table 1:** Comparison of published studies of gut microbiome in PSC

*Study (publication year)*	*Material*	*Cohort*	*Method (region amplified)*	*Diversity*	*Taxa alteration*
Kummen *et al.* ^161^	Stool	85 PSC (55 PSC-IBD)36 UC263 HC	16s rRNA sequencing (V3–V4)	vs. HC↓ vs. IBD↔	*Veillonella*↑
Rühlemann *et al.* ^159^	Stool	73 PSC (38 PSC-IBD)88 UC98 HC	16s rRNA sequencing (V1–V2)	—	*Veillonella*↑ (but not PSC-specific)
Sabino *et al.* ^160^	Stool	52 PSC (39 PSC-IBD)13 UC, 30 CD52 HC	16s rRNA sequencing (V4)	↓	*Enterococcus*, *Lactobacillus and Fusobacterium*↑
Iwasawa *et al.* ^162^	Stool	13 pediatric PSC15 UC23 HC	16s rRNA sequencing (V1–V2)	vs. HC↓ vs. IBD↑	*Enterococcus*↑, *Parabacteorides*↓
Torres *et al.* ^155^	Mucosa	20 PSC (19 PSC-IBD)13 UC, 2 CD9 HC	16s rRNA sequencing (V3–V4)	↔	*Barnesiellaceae*, *Blautia*↑
Quraish *et al.* ^156^	Mucosa	11 PSC-IBD10 IBD9 HC	16s rRNA sequencing (V3–V4)	—	*Escherichia*, *Lachnospiraceae*, *and Megasphera*↑
Rossen *et al.* ^157^	Mucosa	12 PSC-IBD11 UC9 HC	16s rRNAmicroarray	↓	Uncultured *Clostridiales Π*↓
Kevans *et al.* ^158^	Mucosa	31 PSC-UC30 UC	16s rRNA sequencing(V4)	vs. UC ↔	vs. UC↔

### Perspectives and future directions

The commensal microbiome utilizes multiple pathways to shape mucosal immunity, and its impact may reach non-mucosal tissues and thus contribute to systemic autoimmunity in genetically susceptible individuals. The currently available techniques to detail the functions of the intestinal microbiome has provided more accurate information regarding the microbial changes in human autoimmune diseases. However, the scenario is complicated by the variability of the intestinal flora over short periods of time, which does not allow a historical perspective of the observed changes years after the initiation of the autoimmune process. Whether the observed changes are the cause or the consequence of the disease (or its treatment) remains enigmatic. Under this circumstance, several hypotheses have been proposed, aiming at explaining the causal link between the commensal microbiota and the development of diseases. Molecular mimicry, in which the gut microbiota may serve as a source of cross-reactive antigens that trigger autoimmune reactions, was postulated many years ago and is still being re-assessed and validated in different models^9,10,96^ Nevertheless, overwhelming data suggest a multifaceted effect of the microbiota on host physiology.^165^ In most cases, a perturbed microbiota may exacerbate the immune disorder in the setting of genetic susceptibility rather than ignite an initial autoimmune attack.

Undoubtedly, larger, longitudinal and well-controlled microbiome-wide studies are expected in the future field of autoimmune diseases, to better understand the potential confounders as well as the importance of the observed differences. For example, the use of samples from monozygotic twin pairs who were discordant for a disease has, to a large extent, excluded differences derived from genetics, location and diet.^122^ In addition, it seems necessary to consider the influence of drug therapy when analyzing the microbiota.^94,152^ It should be noted that the study of the microbiome has begun to shift from merely describing the association between commensal communities and diseases to exploring the detailed immunologic and metabolic function of specific microbes and, further, seeking for adjuvant therapeutics based on fecal microbiota transplantation (FMT) or application of microbiota-derived bioactive molecules such as probiotics and prebiotics,^166,167^ which hold great promise for future clinical improvements of autoimmune disorders as well as inflammatory diseases.

Although mouse models have now been extensively used to interpret the contribution of colonization by a single microbe, it should be taken into consideration that the human and murine intestinal microbiota have substantial differences.^168^ Moreover, conclusions should be made with caution because the rearing facilities and genetic backgrounds of animal models have been shown to impact their microbial composition and the disease phenotype,^163,164^ potentially leading to poor repeatability in microbiome-associated experiments. Another issue is the age window of dysbiosis-induced autoimmunity, which has been emphasized in several diseases.^109,169^ Consistently, a large-cohort, prospective study has demonstrated that probiotic supplementation as early as 0–27 days is correlated with a reduced risk of islet autoimmunity,^170^ highlighting the effective of the timing of microbiota-associated interventions. Finally, the majority of the current data are gathered from the intestinal microbiota, but other skin and mucosal sites, as well as viruses and fungi, have important roles in the microbiota, underscoring the level of complexity that will need to be solved in the future.^171,172^
